# Analysis of the thermoelectrical performance of samples made of Coir Agricultural Wastes combined with MWCNT

**DOI:** 10.1038/s41598-022-20801-8

**Published:** 2022-10-08

**Authors:** Gustavo Vera-Reveles, Jorge Simón, Edgar Briones, José Manuel Gutiérrez-Hernández, Francisco J. González, Gabriel González, Edmundo Cerda-Rodríguez, José Vulfrano González-Fernández

**Affiliations:** 1 Departamento de Ciencias Básicas, Instituto Tecnológico de San Luis Potosí, Tecnológico Nacional de México, 78437 San Luis Potosí, Mexico; 2 Departamento de Eléctrica, Electrónica y Mecatrónica, Instituto Tecnológico de San Luis Potosí, Tecnológico Nacional de México, 78437 San Luis Potosí, Mexico; 3 Centro de Investigación, Innovación y Desarrollo en Telecomunicaciones, Universidad Autónoma de Zacatecas, 98000 Zacatecas, Mexico; 4 Departamento de Matemáticas y Física, Universidad Jesuita de Guadalajara, 45604 Jalisco, Mexico; 5 Departamento Físico Matemático, Universidad Autónoma de San Luis Potosí, 78000 San Luis Potosí, Mexico; 6 Centro de Ciencia y Tecnología de Terahertz, Universidad Autónoma de San Luis Potosí, San Luis Potosí, 78000 México; 7 Laboratorio Nacional de Ciencia y Tecnología de Terahertz, Universidad Autónoma de San Luis Potosí, 78000 San Luis Potosí, Mexico; 8 Instituto Tecnológico de San Luis Potosí, Tecnológico Nacional de México, 78437 San Luis Potosí, Mexico

**Keywords:** Materials science, Nanoscience and technology

## Abstract

A biomaterial made of coir and Multi-Walled Carbon Nanotubes (MWCNTs) is presented which exhibits a relatively high-Temperature Coefficient of Resistance (TCR) and thermal insulation properties. Bolometers usually offer acceptable thermal isolation, electrical resistance, and high TCR. Fibers from agricultural waste materials such as coir has a synergistic effect as thermal insulating material and noise reducer. Based on it, powdered coir pills were used as pilot samples, as well as 2 other samples with different dispersions of MWCNTs, sodium dodecyl benzene sulfonate (SDBS) and polyvinylpyrrolidone (PVP) solution. The 3 kinds of samples were thermo-electrically characterized to determine their bolometric performance. Thermal conductivity of k = 0.045 W/m K was obtained by solving the Fourier’s law substituting the data into the equation describing heat flux on the sample around room temperature. Results show that adding different concentrations of MWCNT to powdered coir will lead to films with lower electrical resistance, therefore the thermal conductivity increases while thermal resistance decreases. Finally, the bolometric performance shows a maximum peak with a relatively high TCR of − 40.4% at a temperature of 300.3 K, this synthesized material outperforms by almost 1 order of magnitude larger than commercial materials. Results in this work also indicate that it is possible to tune bolometric parameters of this kind of samples and to use them as thermal insulators in the construction industry, when building roofs and walls.

## Introduction

Conductive MWCNTs have been used as conductive fillers in a polymeric matrix such as epoxy/MWCNTs to enhance electrical properties as conductivity, Conductive MWCNTs can also be used as temperature sensors in a wide range of temperatures with relatively acceptable stability and linear characteristics^1,2^.

The increase in heat derived from climate change is melting glaciers and sea ice, shifting precipitation patterns, and setting animals on the move. In fact, since 1906, the global average surface temperature has increased by more than 1.6 °F (0.9 °C)^3^, according to the National Oceanic and Atmospheric Administration (NOAA). In addressing the above-mentioned problems, it is known that thermal insulation can mitigate high temperatures, including those derived from climate change, an example of which are materials based on natural fibers with high porosity^4^. There are several types of materials that are commercially available as sound absorbers and heat insulators, these synthetic materials are hazardous to human health^5^. Agricultural wastes such as coir are non-hazardous natural sources of loss carbon that can be used to absorb electromagnetic waves^6–9^. Agricultural waste is formed by organic compounds from plants and whose main element is carbon, an element which is suitable for converting microwave energy into thermal energy. Among the most important properties of coir, it can be highlighted that it is abundant, non-toxic, biodegradable, low density, and low cost^10^. It is worth mentioning that Mexico is a leader in Latin America producing this agricultural waste, being in the state of Guerrero the greatest production of more than 4,000,000 tons and from which large amounts of coir can be used in the manufacture of products^11^.

The thermoelectric behavior of the coir regarding electrical resistance and temperature for a short period of time shows excellent bolometric properties, a bolometer absorbs incident radiation, which causes a change in its temperature, and due to this temperature change, the electrical resistance of the active material increases or decreases, depending on the alpha value or temperature coefficient of resistance (α or TCR), for this study, the electrical resistance decreases, behaving like a semiconductor material^12^. On the other hand, thermal conductivity is defined as the time rate of steady-state heat flow through a unit area of a homogeneous material induced by a unit temperature gradient in a direction perpendicular to that unit area^13^.

Previous works reported that the composition of a carbon nanotube membrane with the non-conductive phase-changing polymer Poly (*N*-isopropylacrylamide) (PNIPAm), achieved a TCR higher than − 40%/K at 300 K, however, it should be considered that these measurements were obtained with temperatures: initial of 2 °C and final of 45 °C, respectively, with equally spaced intervals of 1 °C^14^. A second work showed that, in the study of uncracked composite films of semi-metal and Single-Walled Carbon Nanotubes (SWCNT), the resistance as a function of temperature (measured at 55 K above the reference temperature) decrease approximately 23% of its value, in an approximate time of 12 min^12^.

In this work, the thermoelectric performance was focused on relatively high TCR and thermal conductivity. About the first term, the TCR reaches a maximum value of − 40.43%/K at 300.3 K, as well as the resistance as a function of temperature (both readings measured in real-time and synchronized) decrease by approximately 23% the resistance value, in 20 s, this value was taken with an increase of only 1 K above the reference temperature. In the second term, the value of the thermal conductivity of the coconut was satisfactorily obtained with a value of k = 0.045 (W/m K) at 300 K by solving the Fourier’s law^12^.

It is worth mentioning that the three coconut samples were dehydrated at the same temperature of 120 °C, for the same time of 24 h.

## Materials and methods

### MWCNT

In this work, crude MWCNT from Thomas Swan Advanced Materials & Co. Ltd. (Durham, UK), the polymer (PVP, 10 kDa) and the surfactant (SDBS) from Sigma-Aldrich, were used respectively. The approximation in the magnitude of the thermal conductivity of the MWCNT is known (“Impact of temperature on the thermal conductivity of Carbon Nanotube (CNT)”), which was taken into account to make the different concentrations that will be added to the powdered coir, which are discussed in “Mixing the coir with MWCNT”.

### Mixing the coir with MWCNT

The powdered coir comes from the state of Colima, Mexico. As a first step in the elaboration of samples, MWCNT powder was mixed with deionized water and SDBS. The solute and solvent were thoroughly combined by sonic agitation provided by a bath-type sonicator during 30 min, after which a polymer surfactant, PVP, was added and the solution was sonicated for an additional time of 5 min^12^.

**Figure 1:**
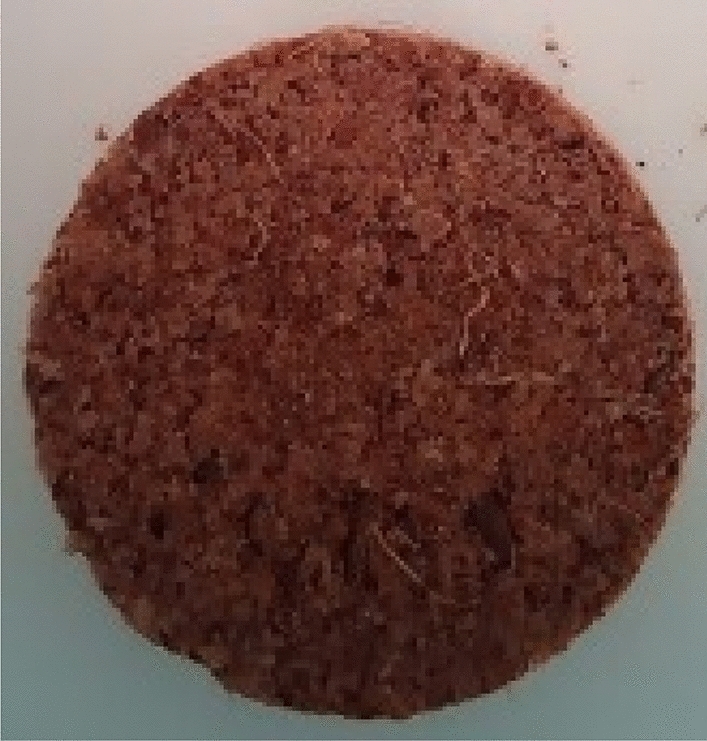
Powdered coir as pills was used as a control sample.

Subsequently, the powdered coir was moistened to make a coir paste using different dispersions of MWCNT-SDBS-PVP solution (two concentrations: 0.125 mg/ml and 0.25 mg/ml). The amounts used for combinations of coir and MWCNT were 1 g of coir and 100 μl of each MWCNT suspensions (0.125 mg/ml and 0.25 mg/ml). Figure 1 shows the coir (1 g) with 100 μl of DI water, prepared as a control. All samples were accomplished by using a manual mixer with a metal tip for 10 min approximately or until obtaining a homogeneous wet mixing. Then, the coir paste was filled into the sample holder, a pressure of 0.5 kPa and heat were applied to dehydrate at 120 °C for 24 h to create coir-MWCNT as pills, whose dimensions were 2.8 cm in diameter and 0.6 cm thick^9^. Two vertical silver stripes were traced with a highly conductive pen (CW2200STP, Chemtronics) and used as electrodes, 2 cm long, 1.2 mm wide, and 1.5 cm apart, to each of the samples separately.

### Impact of temperature on the thermal conductivity of Carbon Nanotube (CNT)

Depending on their structure, form, and synthesis method, thermal conductivity in CNT varies significantly, from 6600 W/m K^15^ for individual SWCNT up to the values that indicate they may even be thermal insulators for MWCNT bundled systems, for which thermal conductivity value is below 0.1 W/m K^16,17^. Yoshida et al.^18^ conducted a research regarding the impact of an increase in the number of films on the reduction in anisotropy and on the increase in thermal conductivity. With further increase in temperature, thermal conductivity starts to be mediated by additional phonon modes and thermal conductivity rises until reaching the maximum value which is often close to room temperature^15,19^. The research conducted by Hone et al., Gonnet et al. and Pӧhls et al.^20–22^ has demonstrated that the films obtained from CNTs had an increase in thermal conductivity as well as an increase in temperature, approximately from 0 K to room temperature, which might have been expected from examination of changes in thermal conductivity for a single nanotube. Hone et al.^20^ were the pioneers in determining the relationship between thermal conductivity and temperature for high-purity mats of tangled single-walled carbon nanotubes. They noticed that the thermal conductivity decreased slowly from 210 to near 0 W/m K with decreasing temperatures in the range from 350 K to below 40 K, respectively. In Ref.^23^, it was analyzed the change in the structure of bulk MWCNTs after annealing and found that the higher the annealing temperature, the higher the density, up to 1.45 g/cm^3^ for the temperature of 2000 °C. The measurements of the thermal properties for disk-shaped MWCNT samples, demonstrated that an increase in annealing temperature, i.e., in density, causes an increase in the value of thermal conductivity from 2.8 to 4.2 W/mK, as well as in the thermal diffusivity.

### Temperature coefficient of resistance

TCR, denoted as α, was obtained from the resistance and temperature of samples as recorded by two independent Fluke 289 digital multimeters connected to a laptop computer. The temperature was measured by a K-type thermocouple while the pill was heated using a Peltier thermoelectric device. The experimental TCR was calculated and defined by Eq. (1)

1 $$ {\text{TCR = }}\frac{{1}}{{\text{R}}} \, \frac{dR}{{dT}} $$

where R is the material electrical resistance at T the operation temperature^24^.

### Heat flow

The heat flow rate was calculated by following the method reported in^12^, defined by:

2 $$ \frac{{{\Delta Q}}}{{{\Delta t}}} = \frac{{\text{L }}}{{\text{R}}}\left[ {\frac{{{\text{T}}_{{1}}^{{2}} }}{{2}} - \frac{{{\text{T}}_{{2}}^{{2}} }}{{2}}} \right] $$

where ΔQ/Δt is the heat production per time, L is the Lorentz number (2.44 × 10^–8^ WΩ/K^2^), R is the electric resistance and finally T_1_ y T_2_ are both temperatures, where T_1_ > T_2_.

### Thermal conductivity

The thermal conductivity (λ) and thermal resistance (R_th_) of the thermal insulation medium were obtained using the data from the measurement of resistance and temperature for each of the samples and their respective physical lengths.

Thermal conductivity was calculated using the formula:

3 $$\lambda =\frac{Ql}{A\cdot \Delta T}$$

where $\lambda$ is the thermal conductivity in W/m K, $Q$ is the heat transfer watts, $\Delta T$ is the temperature difference in Kelvin, $l$ is the thickness in meters, and A is the cross-section area in square meters.

Using the properties to obtain the uncertainty in the measurement^25^, it was developed for each of the terms of the thermal conductivity (Eq. 3), the percentage error of the thermal conductivity was obtained, which is located to the right of the $\pm$ sign, where the term $\updelta$ represents the error measure.

4 $$\lambda =\frac{Q l}{A\cdot \Delta T}\pm \left(\frac{l \delta Q+Q \delta l}{lQ}+\frac{2A \delta T+\Delta T \delta A}{A\Delta T}\right)$$

### Thermal resistance

The R_th_ which is a function of the actual thickness of the material and the thermal conductivity λ was calculated using:

5 $${R}_{th}=\frac{L}{\lambda }$$

where L is the actual thickness of the material expressed in meters^4^.

## Results and discussion

The thermoelectric measurements were performed in real-time and synchronized by monitoring the resistance as a function of temperature for each of the three samples characterized in this work, where it is possible to observe that the control sample has the highest electrical resistance, which decreases when the concentration of MWCNT is increased.

**Figure 2:**
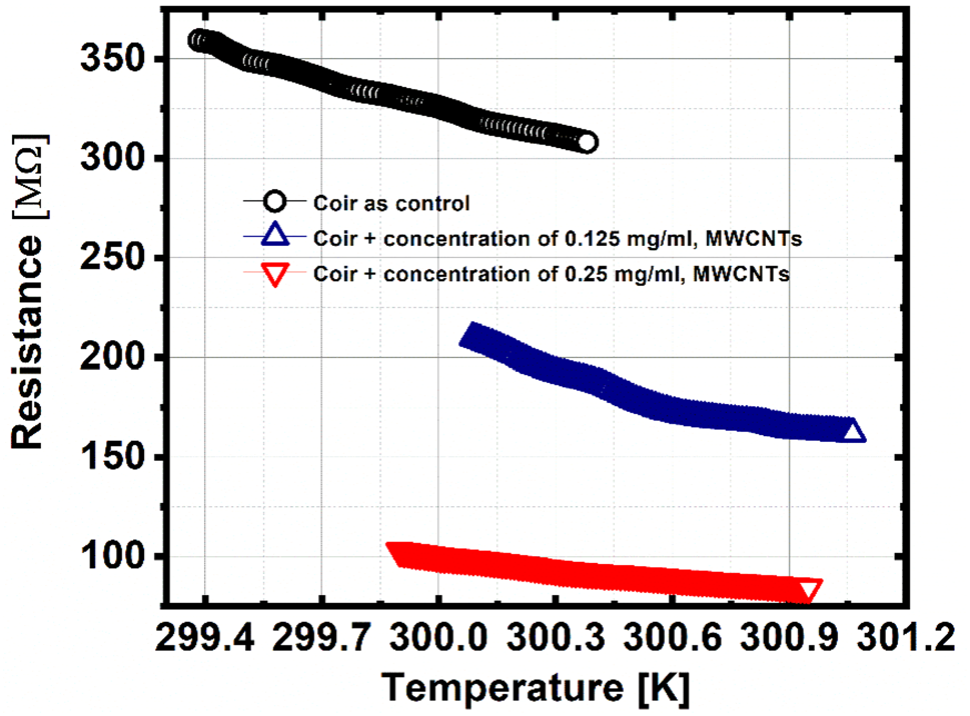
Electrical resistance as a function of temperature.

**Figure 3:**
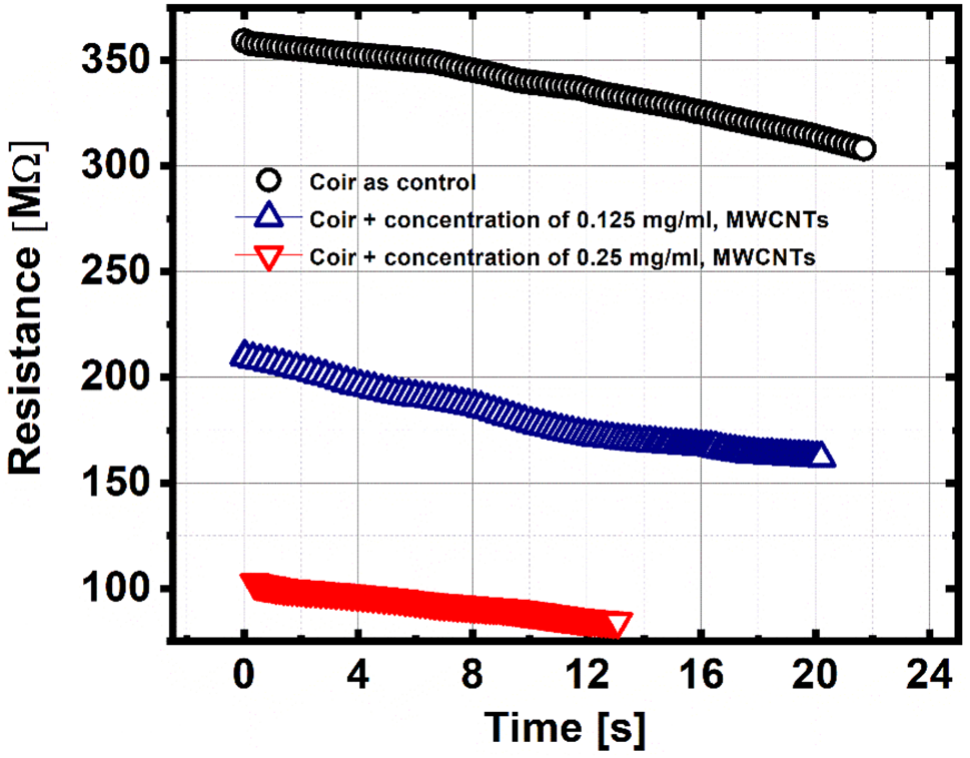
Electrical resistance in real-time when the temperature of the samples increases.

In relation to Figs. 2 and 3, for coir as a pilot sample: when the temperature increases, the electrical resistance decreases approximately 15% of its maximum value of 359.3 MΩ, in a time of 22 s, with a mean TCR of − 15.5%/K. For the coir sample + 0.125 MWCNTs, when the temperature increases, the electrical resistance decreases approximately 23% of its maximum value of 211.7 MΩ, in a time of 20 s, with a mean TCR of − 26.5%/K. Finally, for the coir sample + 0.25 MWCNTs, when the temperature increases, the electrical resistance decreases approximately 18% of its maximum value of 103.36 MΩ, in a time of 13 s, with a mean TCR of -18.7%/K (See Supplementary Information about the decrease in electrical resistance (%) and the time to take place). It is worth mentioning that all the samples were measured at 1 K over a reference temperature. Likewise, it is evident that for the plots in Figs. 3, 6 and 7, a similar process occurs as in Fig. 2, whether in resistance, thermal conductivity and thermal resistance, the samples keep a percentage of reduction or increase very close, concerning the initial base value.

**Figure 4:**
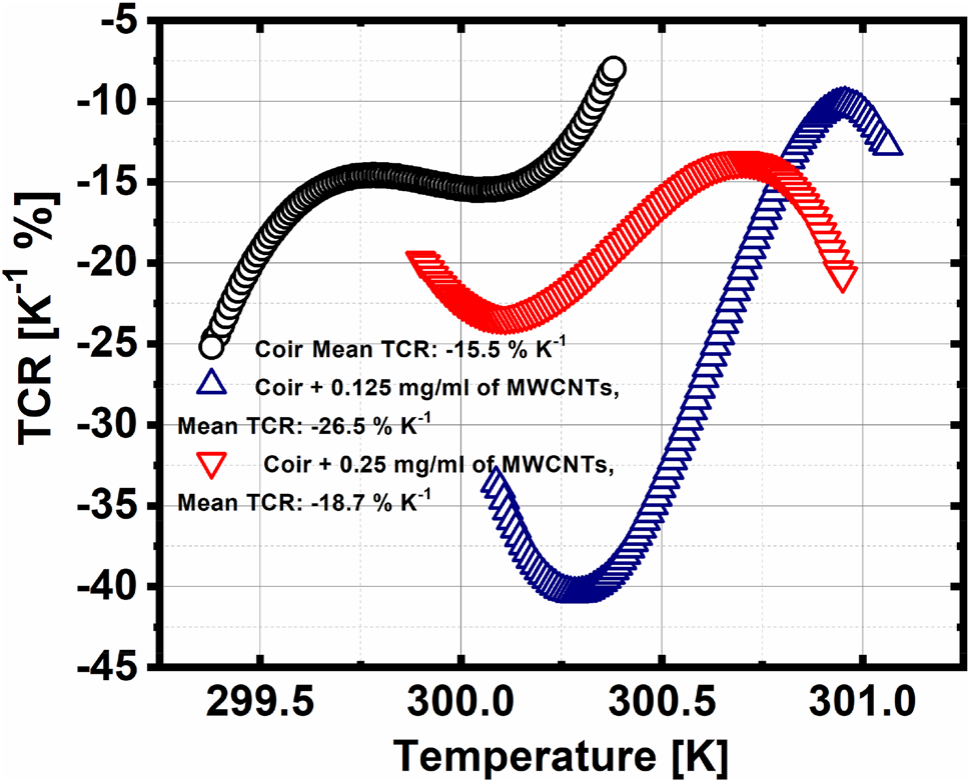
TCR as a function of temperature.

Figure 4 shows TCR as a function of Temperature, the maximum peak in TCR is obtained for the coir sample + 0.125 mg/ml of MWCNTs, being the temperature to reach longer compared to the other two samples.

Tables 1 and 2 show the maximum value of TCR for each of the characterized samples, especially the coir + 0.125 mg/ml of MWCNT sample, which has higher values of TCR and SD than the other two samples. The temperature at which each of the peaks are obtained, is very close to 300 K for the last two samples. In addition, it is worth mentioning that the different values of the maximum TCR value are given, within the first 5 s of having started the thermoelectric characterization of each sample respectively.

**Table 1:** Comparison of bolometric materials and TCR.

Literature	Bolometric material	Absolute TCR (%K^-1^)
Trevor J. Simmons et al. (2015)^12^	SWCNT	6.5
Guadalupe García-Valdivieso (2017)^26^	Thymine, MWCNT	5.6
Enes Battal (2014)^27^	Zinc Oxide	10.4
Gustavo E. Fernandes (2013)^14^	CNT with PNIPAm	40
Current work*	Coir + MWCNT	40.43

*The current work reports perhaps the highest value found in TCR as bolometric response reported to date.

**Table 2:** Shows the temperature at which the maximum TCR is obtained in each of the samples.

Sample	Maximum TCR (% K^−1^)	Mean	SD	Temperature at which TCR is maximum (K)
Coir as control	− 24.71	− 15.51	3.14	299.38
Coir + 0.125 mg/ml of MWCNTs	− 40.43	− 26.57	11.54	300.28
Coir + 0.25 mg/ml of MWCNTs	− 23.37	− 18.71	3.44	300.11

**Figure 5:**
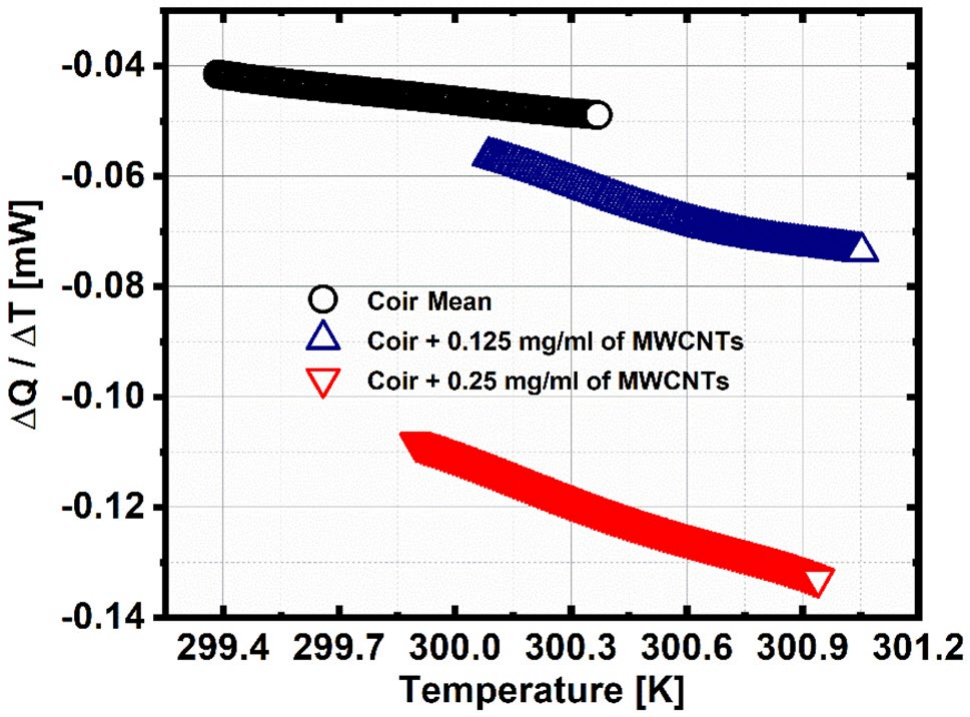
Heat flow as a function of temperature.

The heat flow can be observed in Fig. 5, which for the coir pilot sample has the lowest range (maximum–minimum) of 7.36 µW, for the coir sample + 0.125 MWCNTs, has a range of 17.2 µW, and finally, for the coir sample + 0.25 MWCNTs, has the largest range of 24.5 µW, it can be seen that for greater electrical resistance, there is less heat flow and vice versa.

**Figure 6:**
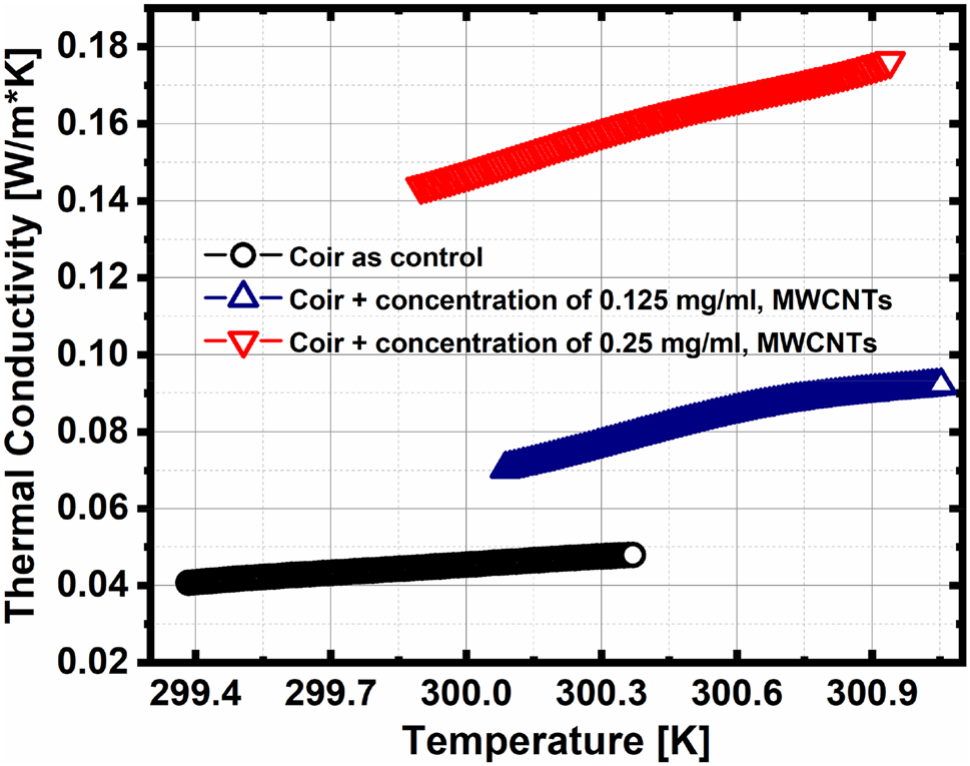
Thermal conductivity as a function of temperature.

The validity of this research is offered by the pilot sample, which agrees with^28–30^; where the thermal conductivity reading is 0.045 W/m K, for a temperature of 300 K. As the concentration of MWCNTs increases, the thermal conductivity also increases (see Fig. 6). This suggests that coir tends to have an improved thermal conductivity performance when combined with other materials, in this case with MWCNT of different concentrations.

**Figure 7:**
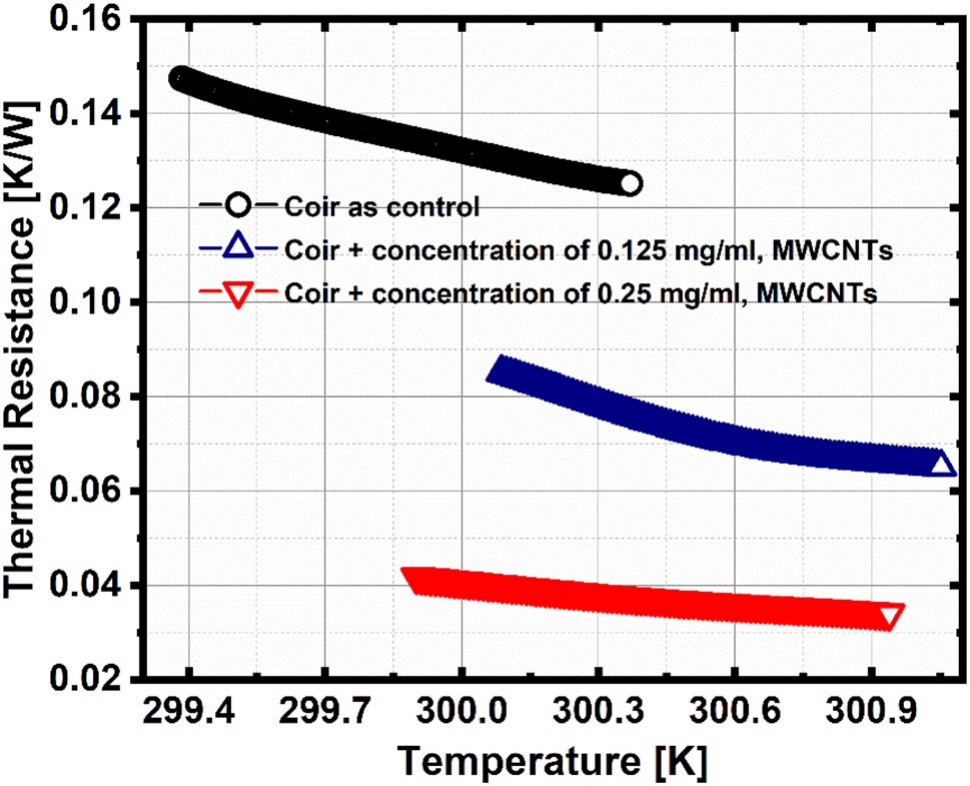
Thermal resistance as a function of temperature.

The thermal resistance is the inverse of the thermal conductivity; therefore, it also has an inverse behavior graphically, a higher thermal resistance corresponds to a lower thermal conductivity, which can be seen in Fig. 7 agreeing with Rodriguez et al.^30^. High thermal resistance assures the effectivity of the developed thermal insulation medium, as well as in Wang et al.^31^, rough surfaced materials are highly porous which has higher thermal resistance resulting in better thermal insulation performance. This further implies that the synergistic effect is evident for coir.

Table 3 shows the thermal characterization means, carried out on the coir samples as pilot samples and coir with MWCNT, at two different concentrations. It is possible to observe that the coir sample with the highest concentration of nanotubes, has the largest heat flux, thermal conductivity, and standard deviation, respectively, compared to the other two samples, as the thermal resistance is the inverse of the thermal conductivity, reason why it has the lowest thermal resistance and standard deviation readings, with respect to the other two samples.

**Table 3:** Shows the mean of heat flow, thermal conductivity, and thermal resistance with their respective SD.

Sample	Heat flow (W) Mean	Standard deviation	Thermal conductivity (W/m K) Mean	Standard deviation	Thermal resistance (K/W) Mean	Standard deviation
Coir as control	− 4.54e − 5	2.13e − 6	0.0445	0.00209	0.135	0.00639
Coir + 0.125 mg/ml of MWCNTs	− 6.65e − 5	5.39e − 6	0.0832	0.00674	0.0725	0.00619
Coir + 0.25 mg/ml of MWCNTs	− 1.21e − 4	7.17e − 6	0.1607	0.00951	0.0374	0.00225

## Conclusions

Based on the knowledge that coir is relatively good absorbing electromagnetic waves, that is why its main element is carbon, which is widely used to transform energy from microwaves into thermal energy, in addition to being a material considered as a thermal insulator, with high electrical resistance and high TCR, the latter reaches a maximum value of − 40.43%/K at 300.3 K, as well as the resistance as a function of temperature decrease approximately 23% the resistance value, in 20 s, this value was taken with an increase of only 1 K above the reference temperature, in the second term, the value of the thermal conductivity of the coconut was satisfactorily obtained with a value of k = 0.045 (W/m K) at 300 K by solving the Fourier’s law; depending on the concentration level of MWCNT, it is possible to tune bolometric parameters of this kind of samples and to use them as thermal insulators in the construction industry, when building roofs and walls. It is concluded that is a material with a performance superior to all the materials whose characterization as bolometer is reported at the moment.

## Supplementary Information

Supplementary Information.

## Data Availability

All data generated or analyzed during this study are included in this published article.
