# Supplementary material for: Analysis of the thermoelectrical performance of samples made of Coir Agricultural Wastes combined with MWCNT
Source: Sci Rep. 2022 Oct 8;12:16935. doi: 10.1038/s41598-022-20801-8 (PMC9547904; doi:10.1038/s41598-022-20801-8)
Supplement: Supplementary file 1 — Supplementary Information. [file 41598_2022_20801_MOESM1_ESM.docx]

**Supporting Information**

Additional images regarding Resistance as a function of Temperature and Time vs Resistance of the three samples characterized in real time, additionally is relevant to indicate that, of the original graphs, an interpolation with 101 points was carried out, to eliminate possible noise peaks, from which a polynomial fit of degree 4 was obtained, substituting the points in each of the equations (1-4), finding smoother graphics without the presence of possible noise from TCR and Heat flow.

**First sample**: Coconut as a pilot sample. When the temperature increases, the electrical resistance decreases by 15% of its maximum value of 359.3 MΩ, in a time of 22 seconds.





Figure S1.





Figure S2.

**Second sample**: coconut + 0.125 MWCNTs

When the temperature increases, the electrical resistance decreases by 23% of its maximum value of 211.7 MΩ, in a time of 20 seconds





Figure S3.





Figure S4.

**Third sample**: coconut + 0.25 MWCNTs

When the temperature increases, the electrical resistance decreases by 18% of its maximum value of 103.36 MΩ, in a time of 13 seconds

*

*

Figure S5.





Figure S6.
